# Neighbourhood socioeconomic deprivation and allostatic load: a multi-cohort study

**DOI:** 10.1038/s41598-019-45432-4

**Published:** 2019-06-19

**Authors:** Ana Isabel Ribeiro, Silvia Fraga, Michelle Kelly-Irving, Cyrille Delpierre, Silvia Stringhini, Mika Kivimaki, Stéphane Joost, Idris Guessous, Martina Gandini, Paolo Vineis, Henrique Barros

**Affiliations:** 1 EPIUnit - Instituto de Saúde Pública, Universidade do Porto, Rua das Taipas, no. 135, 4050-600 Porto, Portugal; 2 Departamento de Ciências da Saúde Pública e Forenses e Educação Médica, Faculdade de Medicina, Universidade do Porto, Porto, Portugal; 3 INSERM, UMR1027, Toulouse, France, and Université Toulouse III Paul-Sabatier, Toulouse, France; 4 Institute of Social and Preventive Medicine, Lausanne University Hospital, Biopôle 2-Route de la Corniche 10, 1010 Lausanne, Switzerland; 5 University College London, Department of Epidemiology and Public Health, London, UK; 6 Clinicum, Faculty of Medicine, University of Helsinki, Helsinki, Finland; 7 Laboratory of Geographic Information Systems (LASIG), School of Architecture, Civil and Environmental Engineering (ENAC), École Polytechnique Fédérale de Lausanne (EPFL), Lausanne, Switzerland; 8 Unit of Population Epidemiology, Division of Primary Care Medicine, Department of Community Medicine, Primary Care and Emergency Medicine, Geneva University Hospitals, Geneva, Switzerland; 9 GIRAPH Lab (Geographic information for research and analysis in public health), Geneva, Switzerland; 10 La Source, School of Nursing, University of Applied Sciences and Arts Western Switzerland (HES-SO), Lausanne, Switzerland; 11 Epidemiology Unit, ASL TO3 Piedmont Region, Grugliasco, (TO) Italy; 12 MRC-PHE Centre for Environment and Health, School of Public Health, Department of Epidemiology and Biostatistics, Imperial College London, London, UK

**Keywords:** Environmental social sciences, Risk factors

## Abstract

Living in deprived neighbourhoods may have biological consequences, but few studies have assessed this empirically. We examined the association between neighbourhood deprivation and allostatic load, a biological marker of wear and tear, taking into account individual’s socioeconomic position. We analysed data from three cohort studies (CoLaus-Switzerland; EPIPorto-Portugal; Whitehall II-UK) comprising 16,364 participants. We defined allostatic load using ten biomarkers of dysregulated metabolic, cardiovascular, and inflammatory systems (body mass index; waist circumference; total, high and low density lipoprotein cholesterol; triglycerides; glucose; systolic and diastolic blood pressure; C-reactive protein). Mixed Poisson regression models were fitted to examine associations with neighbourhood deprivation (in quintiles, Q1-least deprived as reference). After adjustment for confounding variables, participants living in the most deprived quintile had 1.13 times higher allostatic load than those living in the least deprived quintile (Relative Risk, RR, for Q2 RR = 1.06, 95% CI 1.03-1.09; Q3 = 1.06, 1.03–1.10; Q4 = 1.09, 1.06–1.12; Q5 = 1.13, 1.09–1.16). This association was partially modified by individual’s socioeconomic position, such that the relative risk was higher in participants with low socioeconomic position (Q5 vs Q1 1.16, 1.11–1.22) than those with high socioeconomic position (Q5 vs Q1 1.07, 1.01–1.13). Neighbourhood deprivation is associated with biological wear and tear, suggesting that neighbourhood-level interventions may yield health gains.

## Introduction

The effect of individual socioeconomic position (SEP) on health outcomes is well-established^1,2^ and low SEP is now considered a major predictor of morbidity and mortality worldwide^3^. More recently, interest in neighbourhood and area effects of socioeconomic circumstances on health has arisen. It has been postulated that both neighbourhood- and individual-level socioeconomic characteristics contribute to health and health disparities. Neighbourhood socioeconomic deprivation is a marker of contextual characteristics and processes that may affect health, including availability of public services and environmental resources^4–6^.

Supporting this idea, an increasing number of studies have shown that residing in more socially and economically deprived neighbourhoods has been associated with increased risk of disease^4,7^ and death^8^, and this association persists after adjustment for individual-level markers of socioeconomic position. At the biological level, several pathways may link neighborhood deprivation to disease^9^, including elevated inflammation^10^, metabolic disturbances^11^, enhanced responses to stress^12^, and higher allostatic load^13^, a biological concept that captures overall wear and tear.

The concept ‘allostatic load’ (AL) was coined in 1990s by McEwen and Stellar^14^ and offers an integrative model on how the exposure to environmental stressors (social and physical) can generate dysregulation across the body’s multiple systems responsible for maintaining a physiological equilibrium (allostasis)^15^. While small-to-moderate amounts of stressor exposure are beneficial, chronic and cumulative adaptation can overstimulate neuroendocrine and immune systems, leading to permanent physiological damage in cardiovascular activity (elevated blood pressure or heart rate) and the metabolic functioning (raised low density lipoprotein (LDL), triglycerides)^15^. To capture the cost of physiological accommodation across various regulatory systems, the AL is typically expressed as a composite index that includes both primary mediators (neuroendocrine hormones responsible for maintaining a physiological equilibrium after stressor exposure, e.g. epinephrine) and secondary outcomes (biomarkers of cardiovascular, metabolic, and immune functioning, e.g. blood pressure)^16,17^. Investigations using indicators of AL found that it is associated with mortality and ill-health and reported that AL has a superior predictive power of disease risk than its individual components^18^.

As previously described, living in deprived areas is often accompanied by the exposure to stressful environmental conditions, that may lead to psychological distress, physiological damage and consequently to higher AL^13^. However, little is known about the connection between neighborhood socioeconomic deprivation and AL, and the few existing papers on this topic did not explore the interactions between individual socioeconomic position (SEP) and neighbourhood deprivation, which is critical for uncovering etiological pathways.

Socio-spatial segregation – that is the territorial separation of socioeconomic groups belonging to a certain society – is a reality in almost every society, so that it is rare for advantaged individuals to live in deprived neighbourhoods and vice-versa^5,19,20^. Yet, the degree of socio-spatial segregation may vary between cities; in fact, there is a 2-fold difference in the levels of segregation between the most and the least divided European cities^20^. This makes it possible to test whether the health effect of neighbourhood deprivation is modified by individual SEP and, in turn, to evaluate the consequences of socio-spatial segregation^5^.

Theoretical models and empirical research suggest that the effect of neighbourhood deprivation may differ according to individual SEP. The ‘collective resources model’ and the ‘deprivation amplification hypothesis’ postulate that living in disadvantaged neighbourhoods is particularly detrimental to low SEP individuals, because they tend be more reliant on the local services and amenities, which are usually worst in those disadvantaged areas^5,9,21^. On the other hand, the ‘relative standing model’ states that low SEP individuals do not benefit from living in advantaged neighbourhoods and, indeed, they will tend to experience worse health because of the difference between their own SEP and the SEP of their neighbours^5^.

We postulated that individuals who live in deprived neighbourhoods might have higher AL than those living in less deprived neighbourhoods, but we assume a greater influence on AL of living in a disadvantaged area for people who are socioeconomically disadvantaged. Thus, using data from three European prospective cohorts, the objective of this study was to examine the association between neighbourhood deprivation and AL after accounting for individual SEP. In addition, we investigated the presence of cross-level interactions between individual SEP and neighbourhood deprivation.

## Results

Tables 1–3 show the selected sociodemographic, health-related behaviours and biological characteristics according to neighbourhood deprivation quintiles. Mean age was 57.8 in CoLaus, 52.9 in EPIPorto and 50.3 years in Whitehall II. The gender distribution differed according to cohort, with a higher proportion of men in Whitehall II (68.7%) and lower in EPIPorto (38.1%). More than half of the participants from EPIPorto and CoLaus presented low education levels, whereas in Whitehall II, low educated individuals represented only 38.4% of the cohort. The proportion of low SEP individuals increases from the least to the most deprived neighbourhoods while the proportion of high SEP people increases from the most deprived to least deprived neighbourhoods. Despite of this, all types of neighbourhoods show some degree of heterogeneity in terms of individual SEP.

**Table 1:** Descriptive statistics of selected sociodemographic, health-related behaviours and biological characteristics according to neighbourhood deprivation quintiles (CoLaus, n = 5064).

Variables	Total (n = 5064)	Q1-least deprived (n = 1086)	Q2 (n = 1019)	Q3 (n = 1017)	Q4 (n = 991)	Q5-most deprived (n = 951)	p-value^a^
**Age** [Mean (SD)]	57.8 (10.5)	57.9 (9.9)	58.2 (10.4)	58.0 (10.7)	56.9 (10.5)	57.7 (11.1)	0.135
**Males**	2357 (46.5)	527 (48.5)	472 (46.3)	455 (44.7)	465 (46.9)	438 (46.1)	0.522
**Marital Status** (married or similar)	2880 (56.9)	677 (62.3)	600 (58.9)	578 (56.8)	553 (55.8)	472 (49.6)	<0.001
**Education** ^b^							
Low	2677 (52.9)	474 (43.6)	521 (51.1)	563 (55.4)	545 (55.0)	574 (60.4)	<0.001
Medium	726 (14.3)	187 (17.2)	172 (16.9)	138 (13.6)	115 (11.6)	114 (12.0)	
High	1661 (32.8)	425 (39.1)	326 (32.0)	316 (31.1)	331 (33.4)	263 (27.7)	
**Alcohol intake** ^b^							
Abstainer	1281 (25.3)	224 (20.6)	240 (23.6)	240 (23.6)	283 (28.6)	294 (30.9)	<0.001
Low	3443 (68.0)	793 (73.0)	712 (69.9)	715 (70.3)	642 (64.8)	581 (61.1)	
High	340 (6.7)	69 (6.4)	67 (6.6)	62 (6.1)	66 (6.7)	76 (8.0)	
**Smoking** ^b^							
Never smokers	2068 (40.8)	452 (41.6)	425 (41.7)	413 (40.6)	379 (38.2)	399 (42.0)	0.002
Former smoker	1903 (37.6)	428 (39.4)	408 (40.0)	375 (36.9)	359 (36.2)	333 (35.0)	
Current smoker	1093 (21.6)	206 (19.0)	186 (18.3)	229 (22.5)	253 (25.5)	219 (23.0)	
**Sedentariness** (yes)^b^	1620 (32.0)	343 (31.6)	357 (35.0)	339 (33.3)	297 (30.0)	284 (29.9)	0.059
**Allostatic load score**	2.88 (2.02)	2.63 (1.96)	2.87 (2.00)	2.91 (2.08)	2.94 (2.00)	3.07 (2.03)	<0.001
**Cardiovascular system score**	0.49 (0.75)	0.47 (0.75)	0.50 (0.77)	0.49 (0.76)	0.48 (0.75)	0.51 (0.75)	0.458
**Metabolic system score**	2.15 (1.61)	1.95 (1.54)	2.13 (1.59)	2.19 (1.63)	2.20 (1.62)	2.29 (1.65)	<0.001
**Inflammation system score**	0.24 (0.43)	0.21 (0.41)	0.24 (0.43)	0.24 (0.42)	0.26 (0.44)	0.28 (0.45)	<0.001

^a^ANOVA or Kruskal–Wallis test for continuous variables and chi-square test for categorical.

^b^Categorization criteria are fully described in the methods section.

**Table 2:** Descriptive statistics of selected sociodemographic, health-related behaviours and biological characteristics according to neighbourhood deprivation quintiles (EPIPorto, n = 2485).

Variables	Total (n = 2485)	Q1-least deprived (n = 505)	Q2 (n = 493)	Q3 (n = 488)	Q4 (n = 536)	Q5-most deprived (n = 463)	p-value^a^
**Age** [Mean (SD)]	52.9 (15.5)	48.8 (15.3)	52.1 (15.4)	54.7 (15.1)	54.9 (15.2)	54.0 (15.7)	< 0.001
**Males**	946 (38.1)	201 (39.8)	191 (38.7)	184 (37.7)	194 (36.2)	176 (38.0)	0.815
**Marital Status** (married or similar)	1683 (67.7)	361 (71.5)	335 (68.0)	324 (66.4)	370 (69.0)	293 (63.3)	0.082
**Education** ^**b**^							
Low	1516 (61.0)	187 (37.0)	257 (52.1)	304 (62.3)	385 (71.8)	383 (82.7)	<0.001
Medium	320 (12.9)	83 (16.4)	79 (16.0)	63 (12.9)	59 (11.0)	36 (7.8)	
High	649 (26.1)	235 (46.5)	157 (31.8)	121 (24.8)	92 (17.2)	44 (9.5)	
**Alcohol intake** ^**b**^							
Abstainer	846 (34.0)	173 (34.3)	176 (35.7)	160 (32.8)	189 (35.3)	148 (32.0)	0.056
Low	1128 (45.4)	254 (50.3)	219 (44.4)	222 (45.5)	228 (42.5)	205 (44.3)	
High	511 (20.6)	78 (15.4)	98 (19.9)	106 (21.7)	119 (22.2)	110 (23.8)	
**Smoking** ^**b**^							
Never smokers	1404 (56.5)	261 (51.7)	266 (54.0)	293 (60.0)	312 (58.2)	272 (58.7)	0.001
Former smoker	504 (20.3)	97 (19.2)	115 (23.3)	103 (21.1)	116 (21.6)	73 (15.8)	
Current smoker	577 (23.2)	147 (29.1)	112 (22.7)	92 (18.9)	108 (20.1)	118 (25.5)	
**Sedentariness** (yes)^**b**^	2029 (81.7)	364 (72.1)	391 (79.3)	414 (84.8)	462 (86.2)	398 (86.0)	<0.001
**Allostatic load score**	2.43 (2.01)	2.27 (1.93)	2.32 (2.00)	2.31 (1.99)	2.60 (2.08)	2.63 (2.04)	0.004
**Cardiovascular system score**	0.46 (0.72)	0.40 (0.68)	0.44 (0.69)	0.43 (0.70)	0.52 (0.75)	0.52 (0.76)	0.001
**Metabolic system score**	1.71 (1.62)	1.64 (1.59)	1.65 (1.58)	1.65 (1.65)	1.82 (1.67)	1.82 (1.60)	0.099
**Inflammation system score**	0.25 (0.43)	0.23 (0.42)	0.24 (0.43)	0.23 (0.42)	0.26 (0.44)	0.30 (0.46)	0.111

^a^ANOVA or Kruskal–Wallis test for continuous variables and chi-square test for categorical.

^b^Categorization criteria are fully described in the methods section.

**Table 3:** Descriptive statistics of selected sociodemographic, health-related behaviours and biological characteristics according to neighbourhood deprivation quintiles (Whitehall II, n = 8815).

Variables	Total (n = 8815)	Q1-least deprived (n = 1831)	Q2 (n = 1857)	Q3 (n = 1768)	Q4 (n = 1735)	Q5-most deprived (n = 1624)	p-value^a^
**Age** [Mean (SD)]	50.3 (6.1)	50.6 (6.0)	50.4 (6.0)	50.0 (6.1)	50.0 (6.2)	50.4 (6.2)	0.070
**Males**	6057 (68.7)	1466 (80.1)	1380 (74.3)	1308 (74.0)	1079 (62.2)	824 (50.7)	<0.001
**Marital Status** (married or similar)	7360 (83.5)	1697 (92.7)	1649 (88.8)	1537 (86.9)	1331 (76.7)	1146 (70.6)	<0.001
**Education** ^b^							
Low	3386 (38.4)	658 (35.9)	698 (37.6)	647 (36.6)	658 (37.9)	725 (44.6)	<0.001
Medium	2323 (26.4)	524 (28.6)	535 (28.8)	514 (29.1)	411 (23.7)	339 (20.9)	
High	3106 (35.2)	649 (35.4)	624 (33.6)	607 (34.3)	666 (38.4)	560 (34.5)	
**Alcohol intake** ^b^							
Abstainer	1715 (19.5)	255 (13.9)	334 (18.0)	281 (15.9)	400 (23.1)	445 (27.4)	<0.001
Low	5704 (64.7)	1307 (71.4)	1218 (65.6)	1191 (67.4)	1067 (61.5)	921 (56.7)	
High	1396 (15.8)	269 (14.7)	305 (16.4)	296 (16.7)	268 (15.4)	258 (15.9)	
**Smoking** ^b^							
Never smokers	3476 (46.7)	777 (49.3)	733 (46.5)	690 (45.8)	650 (45.3)	626 (46.5)	<0.001
Former smoker	2880 (38.7)	657 (41.7)	644 (40.8)	612 (40.6)	540 (37.6)	427 (31.7)	
Current smoker	1088 (14.6)	143 (9.1)	201 (12.7)	206 (13.7)	246 (17.1)	292 (21.7)	
**Sedentariness** (yes)^b^	1873 (21.2)	214 (11.7)	272 (14.6)	324 (18.3)	462 (26.6)	601 (37.0)	<0.001
**Allostatic load score**	2.45 (2.14)	2.30 (2.10)	2.44 (2.11)	2.46 (2.11)	2.47 (2.20)	2.58 (2.21)	<0.001
**Cardiovascular system score**	0.47 (0.74)	0.44 (0.71)	0.48 (0.75)	0.46 (0.74)	0.49 (0.75)	0.50 (0.76)	0.031
**Metabolic system score**	1.72 (1.69)	1.64 (1.67)	1.73 (1.66)	1.75 (1.67)	1.71 (1.73)	1.79 (1.72)	0.025
**Inflammation system score**	0.25 (0.43)	0.22 (0.42)	0.23 (0.42)	0.25 (0.43)	0.27 (0.44)	0.29 (0.46)	<0.001

^a^ANOVA or Kruskal–Wallis test for continuous variables and chi-square test for categorical.

^b^Categorization criteria are fully described in the methods section.

In the overall sample, the more deprived the neighbourhoods, the higher the AL score. In CoLaus, the AL score ranged from 2.63 in the Q1-least deprived to 3.07 in the Q5-most deprived neighbourhood. Similar patterns were observed in Whitehall II (Q1 = 2.30 and Q5 = 2.58) and in EPIPorto (Q1 = 2.27 and Q5 = 2.63). System-specific scores (cardiovascular, metabolic and inflammatory) also increased with increasing neighbourhood deprivation. Yet, in CoLaus the score representing dysregulation of the cardiovascular system and, in EPIPorto the scores related with the metabolic and inflammation system, despite increasing with neighbourhood deprivation, were not different according to neighbourhood deprivation quintiles.

Health-related behaviours, such as smoking, heavy alcohol consumption and sedentariness were, in general, more prevalent among participants residing in the more deprived neighbourhoods, and the proportion of low educated individuals increased with neighbourhood deprivation.

**Figure 1:**
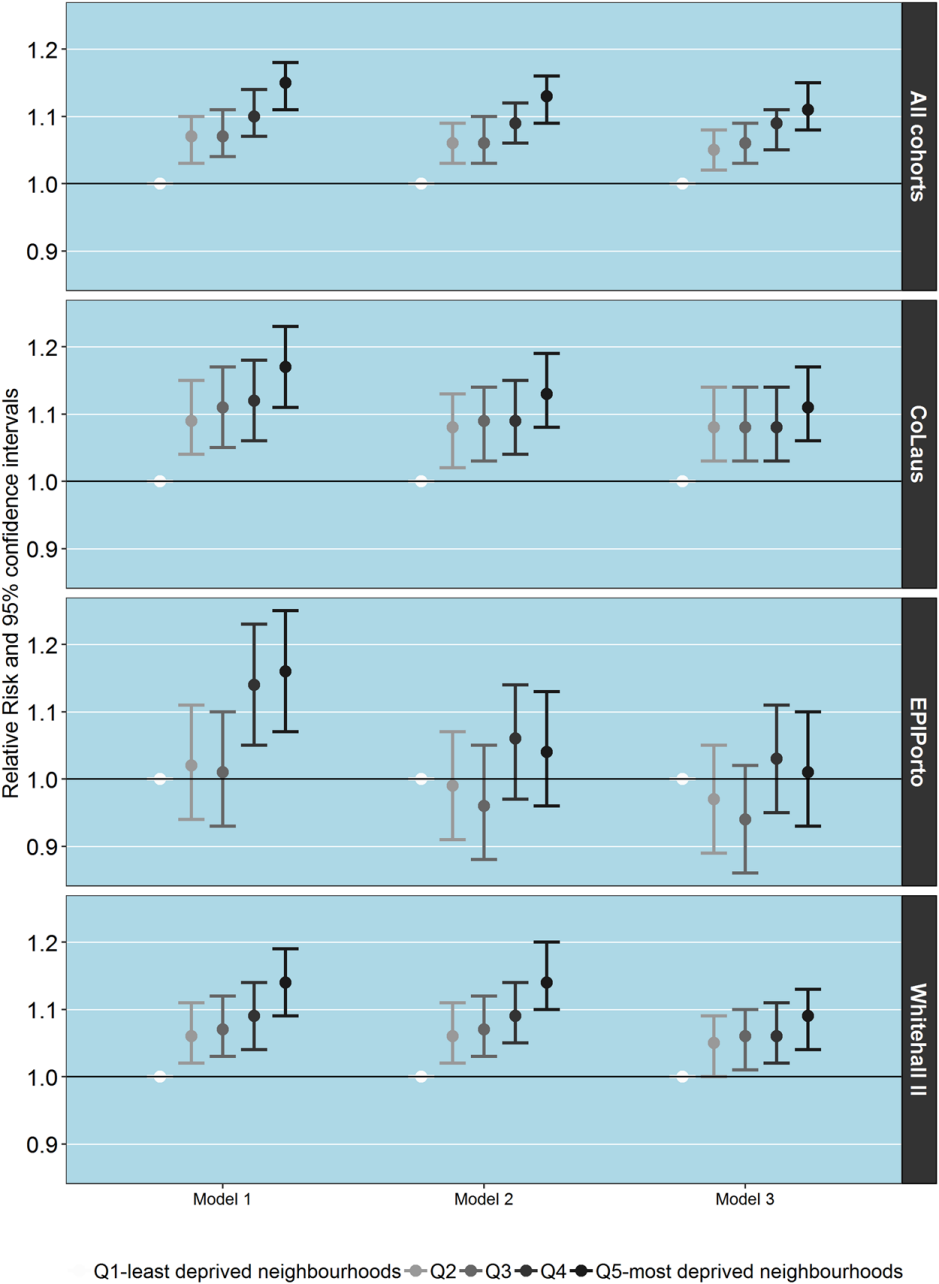
Associations between allostatic load and neighbourhood socioeconomic deprivation according to cohort. Model 1-Adjusted for demographics; Model 2-Adjusted for demographics and individual socioeconomic position; Model 3-Adjusted for demographics, individual socioeconomic position and behaviours.

Figure 1 shows the associations, relative risks, between neighbourhood deprivation (using the least deprived neighbourhoods as references) and AL after adjusting for 1) demographics (age, gender, and marital status); 2) individual SEP (education); and 3) health-related behaviours (smoking, alcohol intake, sedentariness). When we took the three cohorts as a whole, we observed that AL increased with neighbourhood deprivation in a graded manner (Q2 = 1.07, 95% CI 1.03–1.10; Q5 = 1.15, 1.11–1.18). The magnitude of the associations was rather similar among the three cohorts: CoLaus (Q2 = 1.09, 1.04–1.15; Q5 = 1.17, 1.11–1.23); Whitehall II (Q2 = 1.06, 1.02–1.11; Q5 = 1.14, 1.09–1.19); EPIPorto (Q2 = 1.02, 0.94–1.11; Q5 = 1.16, 1.07–1.25).

Adjustment for individual SEP slightly attenuated this gradient (Q2 = 1.06, 95% CI 1.03–1.09; Q5 = 1.13, 1.09–1.16 for the three cohorts as a whole). After accounting for individual SEP, associations between AL and neighbourhood deprivation remained practically unchanged in Whitehall II and CoLaus, but disappeared in EPIPorto. Finally, after adjustment for health-related behaviours, associations were slightly reduced but in Whitehall II and CoLaus most remained.

**Figure 2:**
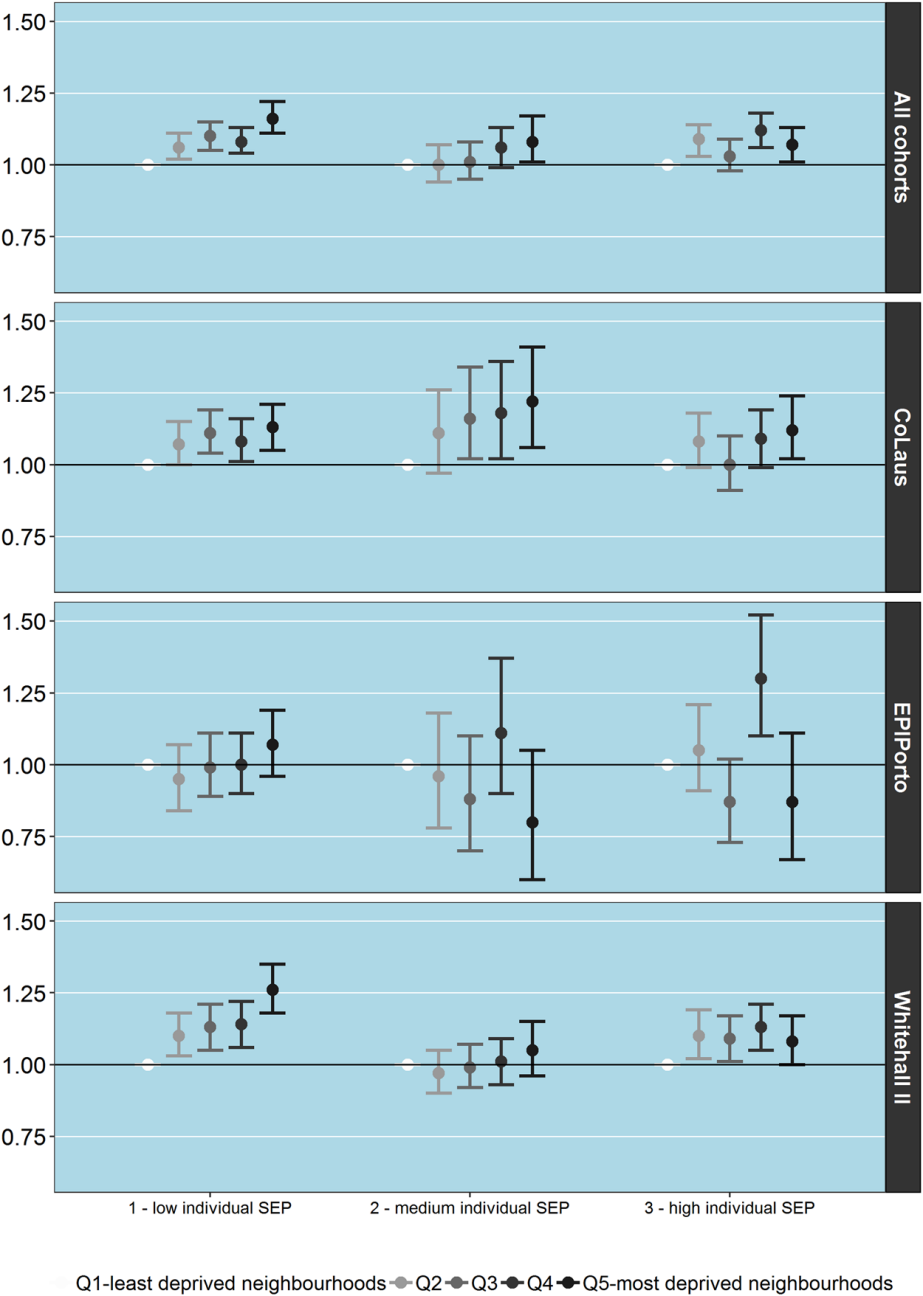
Associations between allostatic load and neighbourhood socioeconomic deprivation according to cohort and stratified by individual socioeconomic position.

Testing for the cross-level interaction between individual SEP and neighbourhood deprivation, revealed there was a interaction between the two, such that the relative risk was higher in participants with low SEP (Q5 vs Q1 1.16, 95% CI 1.11–1.22) than those with high SEP (Q5 vs Q1 1.07, 1.01–1.13, p-value for interaction = 0.024) (Fig. 2).

Yet, cohort differences were observed. The interaction between individual and neighbourhood deprivation was present only in the Whitehall II cohort (p-value for interaction = 0.003) and EPIPorto cohort (p-value for interaction = 0.001). No interactions were observed in CoLaus. As depicted in Fig. 2, in the Whitehall II cohort, the association between neighbourhood deprivation and AL was stronger among low SEP individuals (Q2 = 1.10, 1.03–1.18; Q5 = 1.26, 1.08–1.36), less steep among high SEP individuals (Q2 = 1.10, 1.02–1.19; Q5 = 1.08, 1.00–1.17) and nearly absent among medium SEP individuals (Q2 = 0.97, 0.90–1.05; Q5 = 1.05, 0.96–1.15). In EPIPorto, although the associations were very weak, the gradient of increasing AL with deprivation was only observable among low SEP individuals. In CoLaus the effect of neighbourhood deprivation is grossly the same across classes of individual SEP.

## Discussion

This study examined the link between an important contextual influence, neighbourhood socioeconomic deprivation, and allostatic load (AL), a measure of biological multi-system dysregulation. We found that individuals residing in more deprived neighbourhoods presented higher AL than those living in less deprived ones and that this association remained after accounting for individual socioeconomic circumstances. Additionally, we found evidence that this effect was stronger among individuals of low socioeconomic position (SEP) and less pronounced among individuals of medium and high SEP. However, some cohort specific associations were observed.

Our results extend single-cohort studies. Fourteen studies on the topic, both cross-sectional and longitudinal, were identified in a recently published literature review^13^. However, these studies were highly heterogeneous in terms of AL assessment (biomarkers and formulas of calculation varied substantially between studies), making results not directly comparable among them and with ours^13,22^. Despite methodological differences, most investigations found a significant assocation between neighbourhood socioeconomic structure and AL, even after adjustment for confounding variables such as individual SEP, gender and age. As such harmonized data were not available for the studied cohorts, we could not explore the potential pathways that may explain the observed association. We were only able to assess to what extent adjustment for well-known health-related behaviours (smoking, alcohol consumption and physical inactivity) affected the measured association. We observed that adjusting for these factors attenuated the observed association, but these behaviours did not fully account for them. Although comparatively fewer studies have examined the pathways that link neighbourhood deprivation and AL, it is plausible that besides health-related behaviours, the neighbourhood social and physical environment, individual stress and anxiety, mediate the observed associations.

Theoretical models suggest that neighbourhood deprivation may affect health by multiple interacting pathways. For example, living in a disadvantaged area could adversely affect health, because advantaged neighborhoods often have a better provision of collective resources (e.g. jobs, recreation, public services) and enjoy cleaner environments (e.g. lower exposure to air and water contaminants, more green space), while poor areas often lack health-promoting resources and are more exposed to pollutants and other environmental hazards (a phenomenon known as environmental injustice)^4^. Furthermore, attitudes, beliefs and social norms that operate at area-level were shown to be related with the social and economic characteristics of the neighbourhoods^4^. In addition, those living in poor areas are more likely to feel stressed contributing to the risk of stress-related morbidity and reduced mental well-being^6^. Confirming the relevance of these pathways, Robinnette and co-authors reported that the associations between AL and neighbourhood deprivation can be for the most part attributed to stress and anxiety and to health-related behaviours, such as poor diet, insufficient physical activity and tobacco use^23^. Contrastingly, others found that the connection between neighbourhood deprivation and AL is explained by perceived environmental disturbances in the neighbourhood (e.g. feelings of unsafety, discrimination and environmental harms such as air pollution), but not by health behaviors^15^. Finally, objectively measured and perceived neighbourhood characteristics of the social and physical environment (e.g. disorder, pollution, lack of safety, etc.) were also shown to be mediating in the relation between AL and neighbourhood disadvantage^24^.

Due to the lack of information on residential physical exposures, we could not assess the mediation effect of the neighbourhood attributes on AL. Nevertheless, it is important to refer that studies conducted in the countries and cities of residence of the included participants suggest that more deprived neighbourhoods have worst physical environments: in Porto, geographical accessibility and quality of green space was significantly lower in the most deprived neighbourhood^25^, and in England, disadvantaged neighbourhoods were found to be more polluted^26^ and to lack health-promoting facilities^27^. Thus, although we could not directly conclude this from our study, it is possible that both behaviours and physical environmental factors explain the observed relation between AL and neighbourhood deprivation.

The results corroborate our initial hypothesis that neighbourhood deprivation is associated with AL, but this relation differs according to individual SEP. We observed that neighbourhood deprivation had a higher toll on AL among individuals with lower SEP but this moderation effect of individual SEP was only observable when taking the three cohorts as a whole, among the population of Whitehall II and in a lesser degree in EPIPorto. This is in accordance with the ‘collective resources model’ and the ‘deprivation amplification hypothesis’, which argue that living in disadvantaged areas is particularly damaging to low SEP individuals, as they are more dependent on social services in the community, which tend to be worst and less available in such neighbourhoods.

After accounting for the confounding effect of individual SEP, we found that the detrimental influence of living in deprived neighbourhoods only remained in CoLaus and Whitehall II cohorts, whereas in EPIPorto the associations were strongly attenuated. It is important to highlight that different neighbourhood deprivation indexes were used and it may partially account for the observed cohort-differences. Nonetheless, the smaller differences between neighborhoods in Porto, after adjusting for individual SEP, deserve to be discussed under other assumptions. Findings from several ecological studies suggest that health inequalities based on deprivation are smaller in southern European cities^28^. This may be due to the lesser socio-spatial segregation in southern European cities^20^, a narrower gradient in health-related behaviours^1^, or buffering social factors^29^, that protect citizens against the harms of living in deprived communities. Indeed, Porto has mixed residential areas, in which social housing complexes are embedded in wealthy neighbourhoods^30^, which may also explain findings in EPIPorto.

There are a number of limitations of the current study. First, the cross-sectional analysis precludes causal interpretation and only a single-time measurement of AL was available, which did not allow us to conduct a longitudinal analysis. Second, we could not fully assess the pathways that link neighbourhood deprivation and AL. Third, education was the single available common marker of SEP with complete information; occupation data was not available for wave 3 in Whitehall II and wave 2 in CoLaus, harmonization of occupation position is not optimal across countries, and a substantial amount of individuals were only classified as retired. Fourth, different cohorts used different indexes of neighbourhood socioeconomic deprivation, limiting our ability to make between-cohort comparisons. Nevertheless, it was recently demonstrated that different deprivation indices, namely EDI and Townsend, perform similarly^31^. Fifth, due to data unavailability, we could not fully take into account residential mobility. Yet, for CoLaus, we conducted a parallel analysis excluding movers but estimates remained unaffected (Supplementary Table 8). Sixth, the areal units that were used differed substantially between the cohorts, which might generate inconsistencies, a feature known as the Modifiable Area Unit Problem (MAUP). In EPIPorto and CoLaus spatial units were relatively small allowing to capture small differences in the local environment, whereas in the Whitehall II the use of electoral ward could potentially “wash away” (gerrymander) some local differences. Finally, the Whitehall II cohort, in contrast to the other two studied cohorts, is limited to individuals who were originally civil servants and therefore not representative of the general population living in London. Yet, there is a wide range of neighbourhoods in all three cohorts allowing us to detect associations with allostatic load. Furthermore, the inclusion of Whitehall II (composed by higher SEP individuals) allowed us to achieve a more socioeconomically balanced population sample.

Our study has important strengths and implications too. We utilized prospective and harmonized data from three well-established cohorts representing different societies. It resulted in a large sample size, which allowed us to generate solid and comparable estimates of the association between neighbourhood deprivation and AL. Moreover, this topic has never been investigated in the included cohorts and countries, contributing to address an important gap in the current knowledge. Strict and validated geocoding methods were employed in these cohorts^5,12,32^ and theoretically and methodologically sound multivariate indexes of deprivation were used to characterize neighbourhood social and economic structure. Several robustness checks were conducted to account for possible methodological bias. Finally, the large sample size allowed us to assess interactions between individual and neighbourhood deprivation, and to explicitly test two interpretative models – the ‘relative stating’ and the ‘deprivation amplification’ model.

In conclusion, we found that neighbourhood socioeconomic deprivation is associated with higher AL particularly among low SEP individuals. Our study also demonstrates that, beyond individual-level socioeconomic factors, where one lives is independently associated with AL, which makes reasonable to think that improvements at neighbourhood-level may lead to important health gains.

## Methods

### Study population

The study included three adult cohorts from the LIFEPATH Consortium comprising a population of 19,526 participants: the CoLaus (Switzerland, Lausanne), the EPIPorto (Portugal, Porto) and the Whitehall II (United Kingdom, London). These different cohorts provide very different socio-historical contexts where the relationship between individual and neighbourhoods socioeconomic circumstances may be different. Furthermore, these cohorts were recruited in countries/cities with different levels of income inequality (Portugal had the highest, followed by the UK and by Switzerland)^33^ and socio-spatial segregation (highest in London)^19,20^. This allowed us to test the presence of cross-national differences in neighbourhood effects. The full description of each cohort is provided in Supplementary Table 1.

For EPIPorto we used baseline information (1999–2003, n = 2485), for CoLaus data from wave 2 (2009–2013, n = 5064), and for Whitehall II data from wave 3 (1991–1993, n = 8815), corresponding to the evaluations with the largest number of available biomarkers and which permitted us to compute the AL score. Therefore, this study included a total of 16,364 individuals.

### Ethics

All the studies were approved by the local or national ethics committees and written informed consent was obtained for all the participants^34^. CoLaus was approved by the Institutional Ethics Committee of the University of Lausanne. Ethics approval for the Whitehall II study was obtained from the University College London Medical School committee on the ethics of human research^35^. The EPIPorto study was approved by the Hospital São João Ethics Committee^36^. Research was conducted in accordance with existing guidelines including the the revised Declaration of Helsinki in its last version of 2013, the convention for the protection of human rights and dignity of human being with regard to the application of biology and medicine: Convention on Human Rights and Biomedicine (Council of Europe, Oviedo, 1997), the recommendation of the committee of ministers to member states in research on biological materials of human origin (2006), the CIOMS guidelines on ethics in biomedical research (2020) and the EU directive 95/46/EC on the protection of individuals with regard to the processing of personal data and on the free movement of such data.

### Data sharing statement

This study uses individual-level information that cannot be made openly available due to confidentiality issues. Those interested in developing scientific research grounded on these data, should make a formal request to the principal investigators of each cohort (https://www.lifepathproject.eu/).

### Individual socioeconomic position (SEP)

Educational attainment was used as an indicator of individual SEP since it offers several advantages: (1) it is stable through time during adulthood; (2) it is correlated with other indicators of socioeconomic position (income, wealth, occupational status); (3) it captures the material and intellectual resources of a person, influencing the likelihood of them engaging in behaviours that may be deleterious to health and of using preventive health services^37^; and (4) it is easier to compare between cohorts and countries.

Educational attainment was grouped in the following classes following the typical framework for organizing information on education in the included countries: (1) primary and lower secondary education (from 7 to 9 years after kindergarten designed to give basic education in languages, mathematics and other subjects, is referred to as ‘low’); (2) higher secondary education (around 4–5 more years, high school diploma level, is considered ‘medium’); and (3) tertiary education (any post-secondary degree, such as bachelor´s, master´s or doctoral degrees, is referred to as ‘high’)^3^. The harmonization procedures of SEP data in these cohorts, as part of LIFEPATH study procedures, are fully described elsewhere^3^.

### Geocoding and neighbourhood socioeconomic deprivation

In CoLaus, addresses were geocoded using QGIS (Quantum GIS Development Team, 2013) with the MMQGIS Python plugin (http://michaelminn.com/linux/mmqgis/) facilitating the use of the Google Maps API^12^. In EPIPorto, addresses were geocoded using ArcGIS Online World Geocoding Service and Google Maps^36^. In Whitehall II, participants were geocoded using postcodes. Then, point-in-polygon overlay operations were conducted to ascertain each participant neighbourhood and the corresponding level of socioeconomic deprivation^36^. Neighbourhood socioeconomic deprivation was measured using different multivariable indexes of socioeconomic deprivation: the Townsend index of deprivation in CoLaus and Whitehall II^38^ and the European Deprivation Index (EDI) in EPIPorto^39,40^ fully described in Supplementary Table 2. Yet, neighbourhood deprivation was categorized in all cohorts according to quintiles of increasing socioeconomic deprivation (Q1-least deprived to Q5-most deprived). Whilst EDI and Townsend were built using different methods, it was recently demonstrated that these two indexes perform similarly in capturing area-level socioeconomic deprivation^31^.

### Biomarkers and allostatic load (AL)

Anthropometrics were obtained with the participant wearing light clothing and no footwear as reported in previous publications^41,42^. Biomarkers were measured at the date of evaluation using fasted blood samples and were analyzed under standardized laboratory procedures reported elsewhere^41,42^. Blood pressure was measured according to standard procedures after an appropriate period of resting^38,39^.

AL was characterized based on the initial definition^43^ with enhancements based on Castagné *et al*.^18^ and included ten biomarkers representing three physiological systems: immune and inflammatory system (C-Reactive Protein, CRP); metabolic system (body mass index, total, HDL and LDL cholesterol, glucose, triglycerides and waist circumference); and the cardiovascular system (systolic and diastolic blood pressure). Only biomarkers common to the three cohorts were included.

To calculate AL, each biomarker was dichotomized into high risk versus low risk according to age (10 year age groups) and sex-specific quartiles^18^. The high-risk quartile was the highest quartile of all biomarkers, except for HDL cholesterol^18^. System-specific AL scores and an overall AL score were computed by summing the number of biomarkers in the high-risk quartile. Accordingly, the AL score could possibly range from 0 to 10.

The AL score was calculated using the most common AL operationalization proposed by Seeman^43^. The score sums the number of AL markers falling in the high-risk quartile in order to facilitate comparisons with the published literature. We used age- and sex-specific quartiles to achieve a sufficient number of individuals for each age-sex group and to compare each individual to what can be considered ‘normal’ for his/her age-sex group. This operationalization of the AL has also been used in previous reports from the LIFEPATH project^18,44^.

### Covariates

Based on relevant epidemiological findings and theoretical considerations, estimates were further adjusted for the following covariates: age, sex, marital status (married or cohabiting and living alone), smoking, alcohol consumption and physical activity. These variables were harmonized for previous investigations within the LIFEPATH project under standard procedures described elsewhere^3^. Marital status, age and sex may influence where people live and therefore the level of neighborhood deprivation they are exposed to, as well as biomarker levels, constituting therefore potential confounding variables^13,45,46^. Smoking, alcohol consumption and physical activity also constitute plausible behavioral pathways between neighbourhood and individual SEP and AL^13,23,46^. Briefly, self-reported smoking was categorized into current, former and never smoker^34^. Alcohol consumption was measured in alcohol units/week; participants were categorized as abstainers (0 units/week), moderate (1–21 units/week for men, 1–14 for women), and heavy (>21 units/week for men, >14 for women) drinkers^34^. Physical activity was expressed as a dichotomous variable indicating whether the person led an active or sedentary lifestyle^34^.

### Statistical analysis

Mixed-effects Poisson regression models were used to estimate the associations (relative risks, RR and 95% confidence intervals, CI) between neighbourhood deprivation and AL. A two-level structure was considered with individuals (level 1) aggregated in cohorts (level 2). The presence of a cohort interaction was tested and results were presented for the each cohort separately and for the three cohorts as a whole.

To account for demographic differences, regression models were adjusted for age, sex and marital status. Then, individual SEP (i.e. educational attainment) was added to the models to assess if inclusion attenuated the previously obtained associations. Finally, previously-mentioned individual-level behaviours (smoking, alcohol consumption, physical activity) were included to test whether these explained the differences in AL across neighbourhood deprivation quintiles.

Subsequently, interactions between individual SEP and neighbourhood deprivation were investigated. Associations were presented as Relative Risks and 95% Confidence Intervals, which express the relative change in AL score in each neighbourhood deprivation quintile, as compared with the reference quintile (Q1-least deprived).

To reduce possible biases caused by missing data and attrition, missing values were imputed for the variables included in the models^18^. We performed a multiple imputation model using chained equations implemented in the R software, more precisely in the package ‘mice’ for missing imputation. This technique allows imputing missing information for several variables at a time, through an iterative process (the chained equations)^47^. Imputed variables and the number of missing data are depicted in Supplementary Table 3, totalizing roughly 5% of the data used in our study.

Analyses were conducted in R 3.1.1. using the ‘lme4’, ‘mice’ and ‘ggplot2’ packages.

### Robustness checks

For the cohorts with information on the unit of aggregation (‘neighbourhood of residence’) – EPIPorto and CoLaus – a random effect at neighbourhood level was also added to the models, accounting for the fact that individuals were nested within neighbourhoods. Previous analyses were reproduced using this additional random effect and results remained mostly unaffected (see Supplementary Table 4).

To guarantee that our results were not driven by the process of multiple imputation, we fitted the models using the original dataset imputation. Results remained mostly unchanged (see Supplementary Table 5).

We also computed associations using a fixed-effect meta-analysis. The results revealed small-to-moderate heterogeneity and associations matched those obtained using Mixed-effects Poisson regression (see Supplementary Tables 6, 7).

Finally, to account for spatiotemporal population dynamics, we repeated the analysis for CoLaus after excluding participants that changed their address between wave 1 and 2. The results were little affected (see Supplementary Table 8).

## Supplementary information

Supplementary Table 1-8
