# Supplementary material for: Neighbourhood socioeconomic deprivation and allostatic load: a multi-cohort study
Source: Sci Rep. 2019 Jun 19;9:8790. doi: 10.1038/s41598-019-45432-4 (PMC6584573; doi:10.1038/s41598-019-45432-4)
Supplement: Supplementary file 1 — Supplementary Table 1-8 [file 41598_2019_45432_MOESM1_ESM.pdf]

Supplementary information

**Neighbourhood socioeconomic deprivation and allostatic load: a multi-cohort study**

Ana Isabel Ribeiro[1,2*], Silvia Fraga[1,2], Michelle Kelly-Irving[3], Cyrille Delpierre[3], Silvia Stringhini[4], Mika Kivimaki[5,6], Stéphane Joost[4,7,8, 9,10], Idris Guessous[8,9], Martina Gandini[11], Paolo Vineis[12], Henrique Barros[1,2]

1- EPIUnit - Instituto de Saúde Pública, Universidade do Porto, Rua das Taipas, nº 135, 4050-600 Porto, Portugal
2- Departamento de Ciências da Saúde Pública e Forenses e Educação Médica, Faculdade de Medicina, Universidade do Porto, Porto, Portugal.
3- INSERM, UMR1027, Toulouse, France, and Université Toulouse III Paul-Sabatier, Toulouse, France
4- Institute of Social and Preventive Medicine, Lausanne University Hospital, Biopôle 2-Route de la Corniche 10, 1010 Lausanne, Switzerland
5- University College London, Department of Epidemiology and Public Health, London, UK
6- Clinicum, Faculty of Medicine, University of Helsinki, Finland
7- Laboratory of Geographic Information Systems (LASIG), School of Architecture, Civil and Environmental Engineering (ENAC), École Polytechnique Fédérale de Lausanne (EPFL), Lausanne, Switzerland
8- Unit of Population Epidemiology, Division of Primary Care Medicine, Department of Community Medicine, Primary Care and Emergency Medicine, Geneva University Hospitals, Switzerland
9- GIRAPH Lab (Geographic information for research and analysis in public health), Switzerland
10- La Source, School of Nursing, University of Applied Sciences and Arts Western Switzerland (HES-SO), Lausanne, Switzerland
11- Epidemiology Unit, ASL TO3 Piedmont Region, Grugliasco (TO), Italy
12- MRC-PHE Centre for Environment and Health, School of Public Health, Department of Epidemiology and Biostatistics, Imperial College London, London, UK

*Corresponding author
Ana Isabel Ribeiro
EPIUnit - Instituto de Saúde Pública, Universidade do Porto, Rua das Taipas, nº 135, 4050-600 Porto, Portugal
Tel: +351 222061820
Fax: +351 222061821
e-mail: ana.isabel.ribeiro@ispup.up.pt

S1 Table. Description of the included cohorts.

| Cohort | Description |
|---|---|
| **CoLaus** | The CoLaus (COhorte LAUSannoise) is a prospective study assessing the clinical, biological and genetic determinants of cardiovascular disease in the city of Lausanne, Switzerland. The initial survey was conducted between 2003 and 2006 and included 6,733 participants aged 35-75 years; wave 2 was conducted 5.5 years later and included 5,064 participants. In each survey, data on SEP, lifestyle, mental status and cardiovascular risk factors was collected by questionnaire and clinical examination (https://www.colaus-psycolaus.ch/). |
| **EPIPorto** | The EPIPorto study was initiated in 1999 and recruited 2,485 adult community dwellers aged 18 years or older in the city of Porto, Northwest of Portugal. Participants were invited to visit the Porto Medical School for an interview, which included a questionnaire on social, demographic, behavioural and clinical data, and a physical examination including blood collection. Since the initial recruitment period there have been two additional waves (wave 2 in 2005-08 and wave 3 in 2013-14) (http://ispup.up.pt/research/research-structures/cohorts/). |
| **WHITEHALL II** | The Whitehall II study was established in 1985 to examine the socioeconomic inequalities in health among 10,308 London-based civil servants (6,895 men and 3,413 women) aged 35–55. Baseline examination (wave 1) took place during 1985–1988, and involved a clinical examination and a self-administered questionnaire containing sections on demographic characteristics, health and lifestyle factors, work characteristics, social support, and life events. The first follow-up took place in 1989-90 and the third one in 1991-93 and included 8,815 participants (https://www.ucl.ac.uk/whitehallII). |

S2 Table. Description of neighbourhood deprivation indexes available in each cohort

| Cohort | Indicator | Data (year) | Geographical Unit | Description |
|---|---|---|---|---|
| CoLaus | Townsend index of deprivation | 2000 | hectometric grid (100*100 m) | Composite index of the following variables: 1.Percentage of households without access to a car; 2.Percentage of households with more than one person per room (overcrowding); 3.Percentage of households not owner-occupied (tenure); 4.Percentage of economically active residents who are unemployed. |
| EPIPorto | EDI European Deprivation Index | 2001 | Census block groups (~600 inhab/area) | Composite index of the following weighted variables: % overcrowded households, % households with no bath or shower, % household with no indoor flushing, % households occupied by non-owners, % women aged 65 or more, % individuals with low education, % individuals in low income occupation, and % unemployed individuals. |
| WHITEHALL II | Townsend index of deprivation | 1991 | Electoral ward (~5500 inhab/area) | Composite index of the following variables: 1.Percentage of households without access to a car; 2.Percentage of households with more than one person per room (overcrowding); 3.Percentage of households not owner-occupied (tenure); 4.Percentage of economically active residents who are unemployed. |

S3 Table. Description of the variables/procedures used for multiple imputation.

| Variable | Missing (n): Colaus | Missing (n): EPIPorto | Missing (n): Whitehall II | Used for imputation |
|---|---|---|---|---|
| Sex | 0 | 0 | 0 | YES |
| Marital Status (2 classes) | 47 | 1 | 509 | YES |
| Individual SEP (3 classes of educational attainment) | 5 | 0 | 542 | YES |
| Neighbourhood SES (quintiles of deprivation) | 196 | 2 | 429 | YES |
| Smoking (3 classes) | 57 | 63 | 998 | YES |
| Alchool Intake (3 classes) | 0 | 74 | 503 | YES |
| Sedentariness (2 classes) | 883 | 60 | 496 | YES |
| Systolic Blood Pressure Quantile (2 classes: 1: >p75; 0: <=p75) | 24 | 173 | 733 | YES |
| Distolic Blood Pressure Quantile (2 classes: 1: >p75; 0: <=p75) | 24 | 176 | 734 | YES |
| C-reactive Protein Quantile (2 classes: 1: >p75; 0: <=p75) | 43 | 880 | 1207 | YES |
| Body Mass Index Quantile (2 classes: 1: >p75; 0: <=p75) | 65 | 46 | 741 | YES |
| HDL Cholesterol Quantile (2 classes: 1: <p25; 0: >=p25) | 22 | 206 | 818 | YES |
| LDL Cholesterol Quantile (2 classes: 1: >p75; 0: <=p75) | 68 | 231 | 951 | YES |
| Total Cholesterol Quantile (2 classes: 1: >p75; 0: <=p75) | 22 | 153 | 793 | YES |
| Triglycerides Quantile (2 classes: 1: >p75; 0: <=p75) | 22 | 168 | 793 | YES |
| Waist Circumference Quantile (2 classes: 1: >p75; 0: <=p75) | 29 | 37 | 819 | YES |
| Glucose Quantile (2 classes: 1: >p75; 0: <=p75) | 23 | 181 | 1206 | YES |

S4 Table. Associations (RR and 95% CI) obtained by included a random effect term at neighbourhood level.

| cohort | neighbourhood | adjustment | RR | INF 95% CI | SUP 95% CI |
|---|---|---|---|---|---|
| CoLaus | Q1-highest SES neighbourhoods | adjusted for demographics | 1 | 1 | 1 |
| CoLaus | Q2 | adjusted for demographics | 1,09 | 1,02 | 1,16 |
| CoLaus | Q3 | adjusted for demographics | 1,1 | 1,04 | 1,18 |
| CoLaus | Q4 | adjusted for demographics | 1,13 | 1,06 | 1,2 |
| CoLaus | Q5-lowest SES neighbourhoods | adjusted for demographics | 1,17 | 1,1 | 1,24 |
| EPIPorto | Q1-highest SES neighbourhoods | adjusted for demographics | 1 | 1 | 1 |
| EPIPorto | Q2 | adjusted for demographics | 1,04 | 0,92 | 1,17 |
| EPIPorto | Q3 | adjusted for demographics | 1,02 | 0,91 | 1,15 |
| EPIPorto | Q4 | adjusted for demographics | 1,13 | 1,01 | 1,27 |
| EPIPorto | Q5-lowest SES neighbourhoods | adjusted for demographics | 1,17 | 1,04 | 1,31 |
| CoLaus | Q1-highest SES neighbourhoods | adjusted for demographics + individual SES | 1 | 1 | 1 |
| CoLaus | Q2 | adjusted for demographics + individual SES | 1,07 | 1,01 | 1,14 |
| CoLaus | Q3 | adjusted for demographics + individual SES | 1,08 | 1,02 | 1,15 |
| CoLaus | Q4 | adjusted for demographics + individual SES | 1,1 | 1,03 | 1,18 |
| CoLaus | Q5-lowest SES neighbourhoods | adjusted for demographics + individual SES | 1,14 | 1,07 | 1,21 |
| EPIPorto | Q1-highest SES neighbourhoods | adjusted for demographics + individual SES | 1 | 1 | 1 |
| EPIPorto | Q2 | adjusted for demographics + individual SES | 1 | 0,89 | 1,12 |
| EPIPorto | Q3 | adjusted for demographics + individual SES | 0,97 | 0,86 | 1,09 |
| EPIPorto | Q4 | adjusted for demographics + individual SES | 1,06 | 0,94 | 1,18 |
| EPIPorto | Q5-lowest SES neighbourhoods | adjusted for demographics + individual SES | 1,06 | 0,94 | 1,19 |
| CoLaus | Q1-highest SES neighbourhoods | adjusted for demographics + individual SES + behaviours | 1 | 1 | 1 |
| CoLaus | Q2 | adjusted for demographics + individual SES + behaviours | 1,07 | 1,01 | 1,14 |
| CoLaus | Q3 | adjusted for demographics + individual SES + behaviours | 1,08 | 1,01 | 1,15 |
| CoLaus | Q4 | adjusted for demographics + individual SES + behaviours | 1,09 | 1,02 | 1,16 |
| CoLaus | Q5-lowest SES neighbourhoods | adjusted for demographics + individual SES + behaviours | 1,12 | 1,05 | 1,19 |

| | | | | | |
|---|---|---|---|---|---|
| EPIPorto | Q1-highest SES neighbourhoods | adjusted for demographics + individual SES + behaviours | 1 | 1 | 1 |
| EPIPorto | Q2 | adjusted for demographics + individual SES + behaviours | 0,98 | 0,88 | 1,1 |
| EPIPorto | Q3 | adjusted for demographics + individual SES + behaviours | 0,94 | 0,85 | 1,05 |
| EPIPorto | Q4 | adjusted for demographics + individual SES + behaviours | 1,03 | 0,92 | 1,14 |
| EPIPorto | Q5-lowest SES neighbourhoods | adjusted for demographics + individual SES + behaviours | 1,03 | 0,92 | 1,15 |

S5 Table. Associations (RR and 95% CI) obtained using the non-imputed dataset.

| **cohort** | **neighbourhood** | **adjustment** | **RR** | **INF 95% CI** | **SUP 95% CI** |
|---|---|---|---|---|---|
| CoLaus | Q1-highest SES neighbourhoods | adjusted for demographics | 1 | 1 | 1 |
| CoLaus | Q2 | adjusted for demographics | 1,09 | 1,03 | 1,15 |
| CoLaus | Q3 | adjusted for demographics | 1,12 | 1,06 | 1,18 |
| CoLaus | Q4 | adjusted for demographics | 1,11 | 1,05 | 1,17 |
| CoLaus | Q5-lowest SES neighbourhoods | adjusted for demographics | 1,17 | 1,1 | 1,23 |
| EPIPorto | Q1-highest SES neighbourhoods | adjusted for demographics | 1 | 1 | 1 |
| EPIPorto | Q2 | adjusted for demographics | 0,99 | 0,88 | 1,11 |
| EPIPorto | Q3 | adjusted for demographics | 1 | 0,89 | 1,13 |
| EPIPorto | Q4 | adjusted for demographics | 1,12 | 1,01 | 1,26 |
| EPIPorto | Q5-lowest SES neighbourhoods | adjusted for demographics | 1,15 | 1,02 | 1,28 |
| Whitehall II | Q1-highest SES neighbourhoods | adjusted for demographics | 1 | 1 | 1 |
| Whitehall II | Q2 | adjusted for demographics | 1,08 | 1,03 | 1,14 |
| Whitehall II | Q3 | adjusted for demographics | 1,11 | 1,05 | 1,16 |
| Whitehall II | Q4 | adjusted for demographics | 1,08 | 1,03 | 1,14 |
| Whitehall II | Q5-lowest SES neighbourhoods | adjusted for demographics | 1,13 | 1,07 | 1,2 |
| All cohorts | Q1-highest SES neighbourhoods | adjusted for demographics | 1 | 1 | 1 |
| All cohorts | Q2 | adjusted for demographics | 1,08 | 1,04 | 1,11 |
| All cohorts | Q3 | adjusted for demographics | 1,1 | 1,06 | 1,14 |
| All cohorts | Q4 | adjusted for demographics | 1,1 | 1,06 | 1,13 |
| All cohorts | Q5-lowest SES neighbourhoods | adjusted for demographics | 1,15 | 1,11 | 1,19 |
| CoLaus | Q1-highest SES neighbourhoods | adjusted for demographics + individual SES | 1 | 1 | 1 |
| CoLaus | Q2 | adjusted for demographics + individual SES | 1,07 | 1,01 | 1,13 |
| CoLaus | Q3 | adjusted for demographics + individual SES | 1,09 | 1,04 | 1,15 |
| CoLaus | Q4 | adjusted for demographics + individual SES | 1,08 | 1,02 | 1,14 |

| | | | | | |
|---|---|---|---|---|---|
| CoLaus | Q5-lowest SES neighbourhoods | adjusted for demographics + individual SES | 1,13 | 1,07 | 1,2 |
| EPIPorto | Q1-highest SES neighbourhoods | adjusted for demographics + individual SES | 1 | 1 | 1 |
| EPIPorto | Q2 | adjusted for demographics + individual SES | 0,95 | 0,85 | 1,07 |
| EPIPorto | Q3 | adjusted for demographics + individual SES | 0,95 | 0,85 | 1,07 |
| EPIPorto | Q4 | adjusted for demographics + individual SES | 1,03 | 0,92 | 1,16 |
| EPIPorto | Q5-lowest SES neighbourhoods | adjusted for demographics + individual SES | 1,02 | 0,9 | 1,14 |
| Whitehall II | Q1-highest SES neighbourhoods | adjusted for demographics + individual SES | 1 | 1 | 1 |
| Whitehall II | Q2 | adjusted for demographics + individual SES | 1,08 | 1,03 | 1,14 |
| Whitehall II | Q3 | adjusted for demographics + individual SES | 1,11 | 1,05 | 1,16 |
| Whitehall II | Q4 | adjusted for demographics + individual SES | 1,08 | 1,03 | 1,14 |
| Whitehall II | Q5-lowest SES neighbourhoods | adjusted for demographics + individual SES | 1,13 | 1,07 | 1,2 |
| All cohorts | Q1-highest SES neighbourhoods | adjusted for demographics + individual SES | 1 | 1 | 1 |
| All cohorts | Q2 | adjusted for demographics + individual SES | 1,07 | 1,03 | 1,1 |
| All cohorts | Q3 | adjusted for demographics + individual SES | 1,09 | 1,05 | 1,13 |
| All cohorts | Q4 | adjusted for demographics + individual SES | 1,08 | 1,04 | 1,12 |
| All cohorts | Q5-lowest SES neighbourhoods | adjusted for demographics + individual SES | 1,12 | 1,09 | 1,17 |
| CoLaus | Q1-highest SES neighbourhoods | adjusted for demographics + individual SES + behaviours | 1 | 1 | 1 |
| CoLaus | Q2 | adjusted for demographics + individual SES + behaviours | 1,05 | 0,99 | 1,12 |
| CoLaus | Q3 | adjusted for demographics + individual SES + behaviours | 1,06 | 1 | 1,13 |
| CoLaus | Q4 | adjusted for demographics + individual SES + behaviours | 1,05 | 0,99 | 1,12 |
| CoLaus | Q5-lowest SES neighbourhoods | adjusted for demographics + individual SES + behaviours | 1,12 | 1,05 | 1,19 |
| EPIPorto | Q1-highest SES neighbourhoods | adjusted for demographics + individual SES + behaviours | 1 | 1 | 1 |
| EPIPorto | Q2 | adjusted for demographics + individual SES + behaviours | 0,95 | 0,84 | 1,07 |
| EPIPorto | Q3 | adjusted for demographics + individual SES + behaviours | 0,9 | 0,8 | 1,01 |
| EPIPorto | Q4 | adjusted for demographics + individual SES + behaviours | 1,01 | 0,9 | 1,13 |
| EPIPorto | Q5-lowest SES neighbourhoods | adjusted for demographics + individual SES + behaviours | 0,98 | 0,87 | 1,1 |
| Whitehall II | Q1-highest SES neighbourhoods | adjusted for demographics + individual SES + behaviours | 1 | 1 | 1 |

| | | | | | |
|---|---|---|---|---|---|
| Whitehall II | Q2 | adjusted for demographics + individual SES + behaviours | 1,05 | 1 | 1,11 |
| Whitehall II | Q3 | adjusted for demographics + individual SES + behaviours | 1,08 | 1,02 | 1,14 |
| Whitehall II | Q4 | adjusted for demographics + individual SES + behaviours | 1,05 | 0,99 | 1,1 |
| Whitehall II | Q5-lowest SES neighbourhoods | adjusted for demographics + individual SES + behaviours | 1,08 | 1,02 | 1,15 |
| All cohorts | Q1-highest SES neighbourhoods | adjusted for demographics + individual SES + behaviours | 1 | 1 | 1 |
| All cohorts | Q2 | adjusted for demographics + individual SES + behaviours | 1,04 | 1 | 1,08 |
| All cohorts | Q3 | adjusted for demographics + individual SES + behaviours | 1,07 | 1,03 | 1,11 |
| All cohorts | Q4 | adjusted for demographics + individual SES + behaviours | 1,06 | 1,02 | 1,1 |
| All cohorts | Q5-lowest SES neighbourhoods | adjusted for demographics + individual SES + behaviours | 1,11 | 1,07 | 1,15 |

S6 Table. Associations (RR and 95% CI) obtained using a fixed-effect meta-analysis.

| cohort | neighbourhood | adjustment | rr | rrlow | rrup | i2 |
|---|---|---|---|---|---|---|
| All cohorts | Q1-least deprived neighbourhoods | adjusted for demographics | 1 | 1 | 1 | |
| All cohorts | Q2 | adjusted for demographics | 1,07 | 1,04 | 1,1 | |
| All cohorts | Q3 | adjusted for demographics | 1,07 | 1,04 | 1,1 | |
| All cohorts | Q4 | adjusted for demographics | 1,11 | 1,08 | 1,14 | |
| All cohorts | Q5-most deprived neighbourhoods | adjusted for demographics | 1,15 | 1,12 | 1,18 | **64.5% (34.3% - 80.9%)** |
| All cohorts | Q1-least deprived neighbourhoods | adjusted for demographics + individual SES | 1 | 1 | 1 | |
| All cohorts | Q2 | adjusted for demographics + individual SES | 1,06 | 1,03 | 1,1 | |
| All cohorts | Q3 | adjusted for demographics + individual SES | 1,06 | 1,03 | 1,09 | |
| All cohorts | Q4 | adjusted for demographics + individual SES | 1,09 | 1,06 | 1,12 | |
| All cohorts | Q5-most deprived neighbourhoods | adjusted for demographics + individual SES | 1,12 | 1,09 | 1,15 | **61.3% (27.3% - 79.4%)** |
| All cohorts | Q1-least deprived neighbourhoods | adjusted for demographics + individual SES + behaviours | 1 | 1 | 1 | |
| All cohorts | Q2 | adjusted for demographics + individual SES + behaviours | 1,06 | 1,02 | 1,09 | |
| All cohorts | Q3 | adjusted for demographics + individual SES + behaviours | 1,04 | 1,01 | 1,07 | |
| All cohorts | Q4 | adjusted for demographics + individual SES + behaviours | 1,07 | 1,04 | 1,1 | |
| All cohorts | Q5-most deprived neighbourhoods | adjusted for demographics + individual SES + behaviours | 1,09 | 1,06 | 1,12 | **56.2% (16.4% - 77.0%)** |

S7 Table. Associations (RR and 95% CI) obtained using a fixed-effect meta-analysis (stratified).

| cohort | neighbourhood | Individual SES | rr | rrlow | rrup | i2 |
|---|---|---|---|---|---|---|
| All cohorts | Q1-least deprived neighbourhoods | low | 1 | 1 | 1 | |
| All cohorts | Q2 | low | 1,07 | 1,02 | 1,11 | |
| All cohorts | Q3 | low | 1,1 | 1,06 | 1,15 | |
| All cohorts | Q4 | low | 1,09 | 1,04 | 1,13 | |
| All cohorts | Q5-most deprived neighbourhoods | low | 1,17 | 1,13 | 1,22 | **70.3% (46.4% - 83.6%)** |
| All cohorts | Q1-least deprived neighbourhoods | medium | 1 | 1 | 1 | |
| All cohorts | Q2 | medium | 1 | 0,94 | 1,07 | |
| All cohorts | Q3 | medium | 1,02 | 0,96 | 1,09 | |
| All cohorts | Q4 | medium | 1,06 | 0,99 | 1,13 | |
| All cohorts | Q5-most deprived neighbourhoods | medium | 1,08 | 1,01 | 1,15 | **56.7% (17.5% - 77.3%)** |
| All cohorts | Q1-least deprived neighbourhoods | high | 1 | 1 | 1 | |
| All cohorts | Q2 | high | 1,09 | 1,04 | 1,14 | |
| All cohorts | Q3 | high | 1,03 | 0,98 | 1,09 | |
| All cohorts | Q4 | high | 1,13 | 1,08 | 1,19 | |
| All cohorts | Q5-most deprived neighbourhoods | high | 1,08 | 1,03 | 1,14 | **49.3% (1.5% - 73.9%)** |

S8 Table. Associations (RR and 95% CI) including only individuals that did not change neighbourhood deprivation quintile between wave 1 and wave 2.

| cohort | neighbourhood | adjustment | rr | rrlow | rrup |
|---|---|---|---|---|---|
| CoLaus | Q1-least deprived neighbourhoods | adjusted for demographics | 1,00 | 1,00 | 1,00 |
| CoLaus | Q2 | adjusted for demographics | 1,10 | 1,02 | 1,19 |
| CoLaus | Q3 | adjusted for demographics | 1,11 | 1,02 | 1,20 |
| CoLaus | Q4 | adjusted for demographics | 1,10 | 1,01 | 1,19 |
| CoLaus | Q5-most deprived neighbourhoods | adjusted for demographics | 1,18 | 1,10 | 1,27 |
| CoLaus | Q1-least deprived neighbourhoods | adjusted for demographics + individual SEP | 1 | 1 | 1 |
| CoLaus | Q2 | adjusted for demographics + individual SEP | 1,08 | 1,01 | 1,17 |
| CoLaus | Q3 | adjusted for demographics + individual SEP | 1,09 | 1,01 | 1,18 |
| CoLaus | Q4 | adjusted for demographics + individual SEP | 1,08 | 0,99 | 1,17 |
| CoLaus | Q5-most deprived neighbourhoods | adjusted for demographics + individual SEP | 1,14 | 1,06 | 1,22 |
| CoLaus | Q1-least deprived neighbourhoods | adjusted for demographics + individual SEP + behaviours | 1 | 1 | 1 |
| CoLaus | Q2 | adjusted for demographics + individual SEP + behaviours | 1,08 | 1,01 | 1,16 |
| CoLaus | Q3 | adjusted for demographics + individual SEP + behaviours | 1,08 | 1 | 1,17 |
| CoLaus | Q4 | adjusted for demographics + individual SEP + behaviours | 1,07 | 0,99 | 1,16 |
| CoLaus | Q5-most deprived neighbourhoods | adjusted for demographics + individual SEP + behaviours | 1,11 | 1,03 | 1,17 |
